# Efficacy and safety of acupuncture therapy for urinary incontinence in women: A systematic review and meta- analysis

**DOI:** 10.1097/MD.0000000000017320

**Published:** 2019-10-04

**Authors:** Dan Zhong, Wenjun Tang, Dan Geng, Chengshi He

**Affiliations:** a Hospital of Chengdu University of Traditional Chinese Medicine; b Sichuan China 81 Rehabilitation Center, Chengdu, China.

**Keywords:** acupuncture, systematic review, urinary incontinence, women

## Abstract

**Background:**

Urinary incontinence (UI), affects women more frequently than men, with a prevalence to 30–40% of perimenopausal women and almost 50% among women aged over 70 years. caused severe psychological burden and bringing negatively impact to the quality of life, increased caregiver burden and economic cost. Acupuncture is often used to treat them. We aim to conduct a systematic review to evaluate the efficacy of acupuncture for women experiencing UI.

**Methods:**

The following electronic databases will be searched from inception to Jan. 2020: Cochrane Central Register of Controlled Trials (CENTRAL), PubMed, Web of Science, EMBASE, China National Knowledge Infrastructure (CNKI), Traditional Chinese Medicine, Chinese Biomedical Literature Database (CBM), Wan-Fang Database and Chinese Scientific Journal Database (VIP database).All published randomized controlled trials in English or Chinese related to acupuncture for urinary incontinence in women will be included. The primary outcome will be the change from baseline in the amount of urine leakage measured by the 1-hour pad test. Adverse events will be the secondary outcome. Study selection, data extraction, and assessment of study quality will be performed independently by two reviewers. RevMan V.5.3.5 software will be used for the assessment of risk of bias and data synthesis.

**Results:**

This study will provide a high-quality synthesis of current evidence of acupuncture for UI from the 1-hour pad test.

**Conclusion:**

The conclusion of our study will provide an evidence to judge whether acupuncture is an effective intervention for patients suffered from UI.

**PROSPERO registration number:**

CRD42019133195

## 1 Introduction

Urinary incontinence (UI), also known as involuntary urination, is defined as any involuntary loss of urine on physical exertion, sneezing, or coughing and characterized by 3 main types: urgency, stress, and mixed. UI affects women more frequently than men, with a prevalence to 30% to 40% of perimenopausal women and almost 50% among women aged over 70 years.^[1,2]^ UI can cause severe psychological burden (social isolation, low self-esteem, depression, etc.) and bringing negatively impact to the quality of life, increased caregiver burden and economic cost.^[2,3]^ There are several significant risk factors of UI among including age, obesity, parity, vaginal delivery, high-impact exercises, menopause, intestinal constipation, gynecological surgeries, chronic diseases, drug use, caffeine consumption, smoking, and medical condition.^[4,5]^

A variety of treatment options exist that can significantly improve UI symptoms include lifestyle modifications, medications, surgical options, etc. Acupuncture is widely used in China and western countries as a complementary and alternative therapeutic technique in various diseases. Acupuncture has been found to decrease urine leakage and may be an effective treatment option for UI.^[6]^ According to the theory of traditional Chinese medicine, UI is mainly resulted from kidneys’ *qi* deficiency, which often causes bladder dysfunction to control urine. Thus, the objective of acupuncture is to reinforce *qi* of kidneys and promote the bladder function recovery.^[7]^

Previous studies found limited results supporting acupuncture as an effective treatment method for UI and there is no systematic review specifically focused on the acupuncture for UI in women. Hence, a comprehensive review is needed to determine whether acupuncture is an effective and safe treatment for UI in women. Herein, we present the protocol for a systematic review and meta-analysis that aims to evaluate the effectiveness and safety of acupuncture therapy for women with UI.

## 2 Methods and analysis

### 2.1 Study registration

This systematic review protocol was registered with PROSPERO 2019 (registration number: CRD42019133195). And the protocol report is in the base of the Preferred Reporting Items for Systematic Reviews and Meta-Analyses Protocols (PRISMA-P) declaration guidelines.^[8]^ The review will be performed in line with the PRISMA declaration guidelines.^[9]^

### 2.2 Inclusion criteria for study selection

#### 2.2.1 Type of study

RCTs of acupuncture therapy for urinary incontinence in women without restrictions on publication status will be eligible for inclusion.

#### 2.2.2 Type of participant

Female participants who were 18 years or older with urinary incontinence will be included in spite of the race, education, or economic status.

#### 2.2.3 Type of intervention

Acupuncture therapy, which includes manual acupuncture, body acupuncture, electroacupuncture, plum blossom needle, fire needling and warm needling. Other methods, including transcutaneous electrical nerve stimulation, laser acupuncture, cupping, dry needling and moxibustion, will be excluded.

Comparison interventions, which include sham acupuncture (including sham acupuncture at selected acupoints, sham acupuncture at non-acupoints, pseudo-acupuncture interventions, needling at inappropriate acupoints and nonpenetrating sham acupuncture), placebo, medication, usual care, no treatment, and other conventional therapies, will be included.^[10]^ In addition, the review of trials evaluating acupuncture combined with another treatment compared with other typical treatments alone will be included.

#### 2.2.4 Type of outcome measure

The change from baseline in the amount of urine leakage measured by the 1-hour pad test will be accepted as the primary outcome. Adverse events will be the secondary outcome.

### 2.3 Search methods for identification of studies

#### 2.3.1 Electronic data sources

The following electronic databases will be searched from inception to Jan. 2020: Cochrane Central Register of Controlled Trials (CENTRAL), PubMed, Web of Science, EMBASE, China National Knowledge Infrastructure (CNKI), Traditional Chinese Medicine, Chinese Biomedical Literature Database (CBM), Wan-Fang Database and Chinese Scientific Journal Database (VIP database). All published randomized controlled trials in English or Chinese related to acupuncture for urinary incontinence in women will be included.

#### 2.3.2 Searching other resources

The reference lists of potentially missing eligible studies will be scanned ant the relevant conference proceedings will be scanned as well.

#### 2.3.3 Search strategy

The search strategy for PubMed is shown in Table 1. The following search keywords will be used: Urinary Incontinence (e.g., “Urinary Incontinence”); Urinary Incontinence, Stress (e.g., “Urinary Incontinence, Stress”); Urinary Incontinence, Urge (e.g., “Urinary Incontinence, Urge”); acupuncture (e.g., “acupuncture” or “acupuncture therapy” or “body acupuncture” or “manual acupuncture” or “electroacupuncture or “fire needling” or “plum blossom needling”; randomized controlled trial (e.g., “randomized controlled trial” or “controlled clinical trial” or “random allocation” or “randomized” or “randomly” or “double-blind method” or “single-blind method” or “clinical trial”. The equivalent search keywords will be used in the Chinese databases.

**Table 1:**
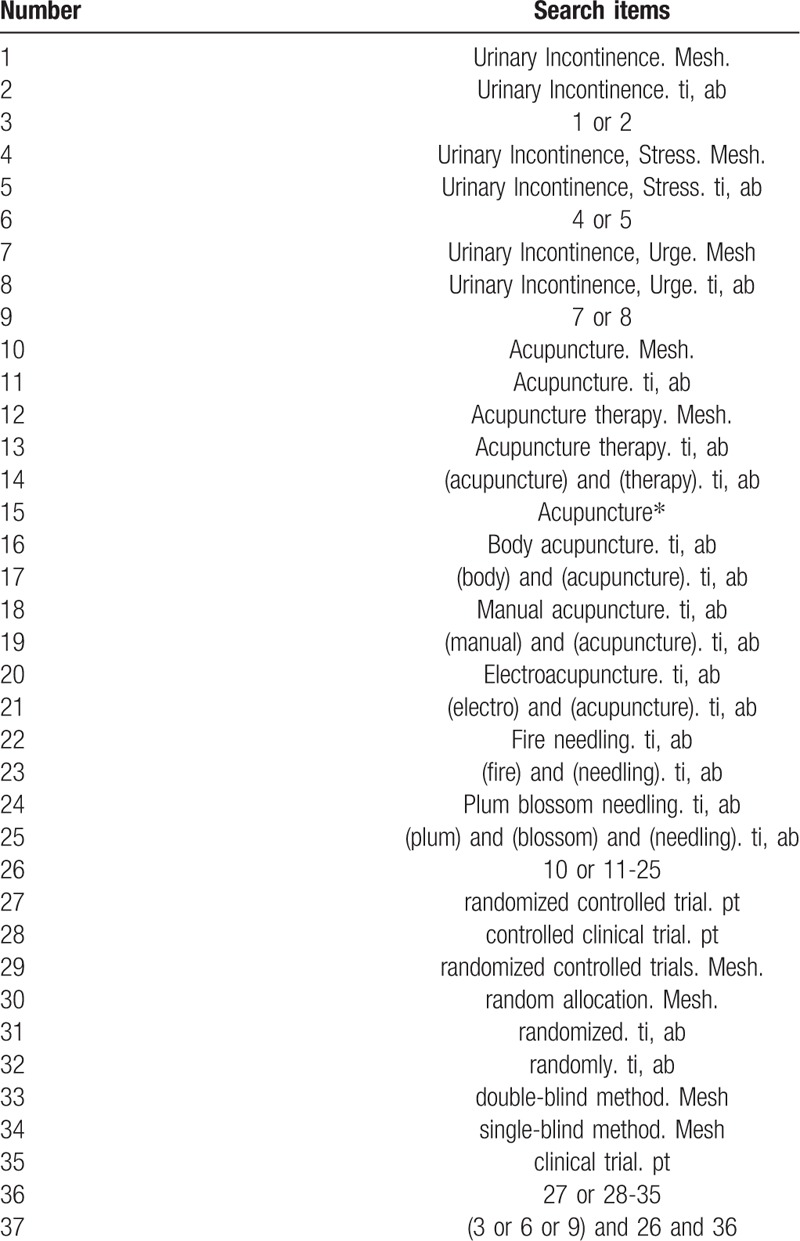
Search strategy for the PubMed database.

### 2.4 Data collection and analysis

#### 2.4.1 Selection of studies

**Figure 1:**
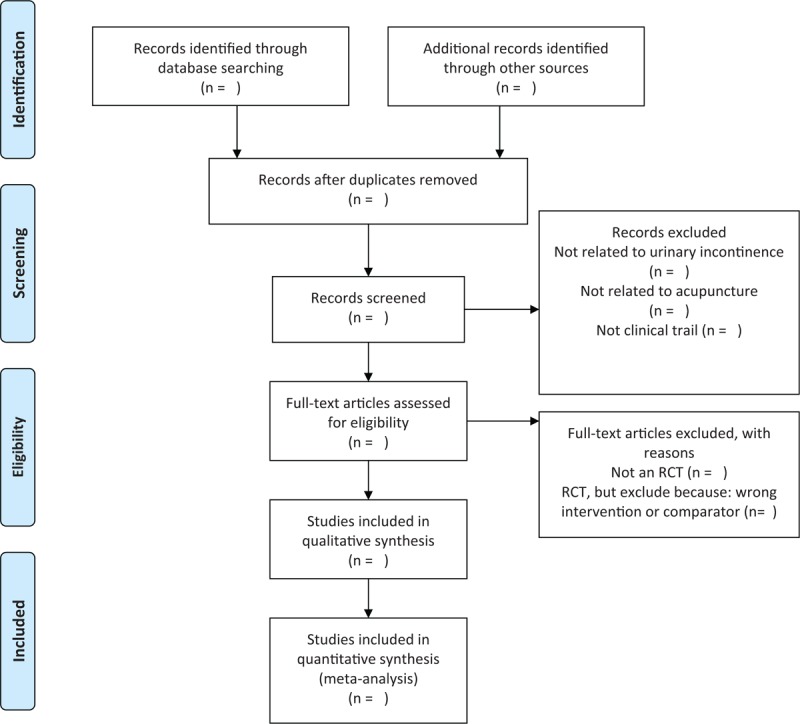
The PRISMA flow chart of the selection process.

The titles and abstracts of all searched studies will be reviewed and screened independently by two reviewers, aiming at identifying eligible trials and eliminating duplicated or irrelevant studies in line with the criteria; the full text of all possibly eligible studies will obtained if necessary. A discussion with the third reviewer is planned to solve the disagreements. A PRISMA flow diagram will be used to show the study selection process (Fig. 1).

#### 2.4.2 Data extraction and management

Before the data extraction, a standard data extraction form with following information will be created: year of publication, general information, country, participant characteristics, inclusion and exclusion criteria, sample size, methods, randomization, blinding methods, type of acupuncture interventions, control, outcome measures, results, conflicts of interest, adverse reactions, ethical approval and other information. And these data will be extracted by 2 independent reviewers. A third reviewer will be set to be discussed with and judge the disagreements during the course. If there is no sufficient data in the publications, the authors will be contacted for further information. Review Manager software (RevMan V.5.3.5) will conduct the analysis and synthesis after transferring all the data into the software.

#### 2.4.3 Assessment of risk of bias in included studies

Two independent reviewers will evaluate the risk of bias in all included studies with the Cochrane Collaboration's tool assessment method and complete the Standards for Reporting Interventions in Clinical Trials of Acupuncture checklist for the studies included.^[11]^ The following domains will be evaluated: selection bias, performance bias, detection bias, attrition bias, reporting bias, and other sources of bias. The assessments will then be divided into 3 levels: low risk, high risk, and unclear. Unclear or insufficient items will be obtained by contacting the corresponding author for further information. The third reviewer will be set for the disagreements.

#### 2.4.4 Measures of treatment effect

Dichotomous data will be presented as risk ratio (RR) and 95% confidence intervals (CI), while continuous outcomes will be showed as standard mean difference (SMD) 95% CI.

#### 2.4.5 Unit of analysis issues

The analytical unit will be the individual participant.

#### 2.4.6 Management of missing data

Contacting the corresponding authors of the included studies will be contacted, including sending emails or making a call, to obtain the missing or insufficient data of the primary results. If missing data is not available, an intent-to-treat analysis will be performed as much as possible (the analysis should include data from all participants in the initially randomly assigned group) and a sensitivity analysis will be performed to determine if the results are inconsistent.

#### 2.4.7 Assessment of heterogeneity

I^2^ test will be applied to quantified inconsistency and standard χ^2^ test will be applied to detect statistical heterogeneity. Studies will be considered to have homogeneity if the *P* value exceeds .1 and the I^2^ value is less than 50%, and the fixed-effects model will be used. While studies will be considered to have significant statistic heterogeneity if the *P* value is less than .1 or the I^2^ value exceeds 50%, and subgroup analysis will be used to explore the possible cause. And the random-effects model will be applied if the heterogeneity is still important.

#### 2.4.8 Assessment of reporting biases

Funnel plots will be used to assess the reporting biases if more than 10 studies are included.

#### 2.4.9 Data synthesis

Review Manager software (RevMan V.5.3.5) will be used to perform the data synthesis if meta-analysis is possible. If no substantial statistical heterogeneity is detected, the data synthesis will be processed with the fixed-effects model, and if substantial statistical heterogeneity is detected, the data synthesis will be performed with the random-effects model. Possible reasons will be searched from a clinical and methodological perspective if different studies exist significant heterogeneity, and descriptive analysis or subgroup analysis will be provided. If there is no substantial heterogeneity between 2 studies, the descriptive analysis will be conducted.

#### 2.4.10 Subgroup analysis

Subgroup analysis will be performed to interpret the heterogeneity if possible. Factors like different acupuncture types and different control interventions will be taken into account.

#### 2.4.11 Sensitivity analysis

Sensitivity analyses will be performed to evaluate the impact of sample size, study design, methodological quality and the effect of missing data, and to verify the robustness of the review conclusions if possible. The analysis will be repeated after low quality studies are excluded.

#### 2.4.12 Grading the quality of evidence

The Grade of Recommendations Assessment, Development and Evaluation (GRADE) will be the tool to evaluate the quality of the evidence.^[12]^ Limitation of study design, inconsistency of results, imprecision, indirectness and publication bias will be assessed. The assessments will be divided into four levels: very low, low, moderate, or high.

## 3 Ethics and dissemination

Formal ethical approval is not necessary for the data cannot be individualized. The results of this protocol will be disseminated in a peer-reviewed journal or presented at relevant conferences. The essential protocol amendments will be documented in the full review.

## 4 Discussion

This systematic review will assess the effectiveness and safety of acupuncture for urinary incontinence in women. The review contains 4 sections: identification, study inclusion, data extraction, and data synthesis. And this will help the doctors to choose acupuncture as an alternative treatment for urinary incontinence in women, and offer the patients more options for their conditions.
